# Diagnosing the context is as important as diagnosing the individual

**DOI:** 10.3389/fpsyt.2025.1698878

**Published:** 2025-10-23

**Authors:** Laura Batstra, Allen Frances

**Affiliations:** ^1^ Department of Child and Family Welfare, University of Groningen, Groningen, Netherlands; ^2^ Department of Psychiatry, Duke University, Durham, NC, United States

**Keywords:** DSM-diagnosis, context diagnosis, social detereminants, inequity and social justice, social prescribing

## Introduction

Many Western countries have experienced a dramatic increase in reported rates of psychiatric diagnoses and use of psychiatric drugs (1–7). The popularity of the third edition of the Diagnostic and Statistical Manual of Mental Disorders (DSM-III), published in 1980, contributed to this, because it greatly increased interest in psychiatric diagnosis (8). By focusing almost exclusively on the troubled individual, DSM’s have diverted attention away from the social context in which mental suffering occurs (9). Our goal here is to first provide a brief summary of the history of modern psychiatric diagnosis and then to indicate why and how individual diagnosis should always be supplemented by context diagnosis. 

## History of modern psychiatric diagnosis

Modern psychiatric diagnosis began with Philippe Pinel (1745–1826), who took a descriptive approach based on careful observation of deviant behaviors and their course (10). Since Pinel, nosographers have devised dozens of diagnostic systems cutting the pie of human distress in a bewildering variety of different ways- none clearly superior to any other. For example (11), Emil Kraepelin in the 19th century introduced a biologically oriented, descriptive classification of mental disorders. The somatoetiological model followed, seeking to ground psychiatric diagnoses in physical causes. During World War II, the U.S. military developed Medical 203 (1945), a practical diagnostic system. This influenced the DSM-I (1952) and DSM-II (1968), both shaped by psychodynamic theory. A shift toward reliability emerged with the Research Diagnostic Criteria (RDC) in the 1970s, culminating in the DSM-III (1980) and its successors, which adopted a criteria-based, atheoretical framework that redefined psychiatric diagnosis. It is fair to say that throughout its history, psychiatric diagnosis has always been over-rated, purporting to explain what it could only describe and maintaining only an incomplete relationship with treatment choice and response.

Before DSM-III’s publication in 1980, psychiatric diagnosis had limited impact on clinical practice. DSM-I (1952) and DSM-II (1968) were thin books mainly used administratively. DSM-III, by contrast, was a thick book that laid out detailed diagnostic criteria and descriptions for each diagnosis. DSM-III claimed to be atheoretical, but its symptom oriented approach clearly favored a biological model over the more inferential psychodynamic model. Although it was not the intention of organized psychiatry to develop a taxonomy that was an industry-friendly instrument, Robert Spitzer, the chair of the DSM III, later acknowledged that “the pharmaceuticals were delighted” with the medical model adopted by the DSM (12). The biomedical model was fueled by significant investments from industry and the government, as well as by advancements in neuroscience and the development of new medications (8, 13). DSM-III gained more legitimacy than it deserved, due to its extensive utilization in public health and administrative contexts, including census taking, statistical reporting, military fitness assessment, and numerous other purposes. It also reshaped both educational curricula and clinical training and its use became a requirement for clinical research funding and insurance payments for treatment (8, 14). DSM-III marked a paradigm shift and transformed psychiatry from broad, etiologically based constructs to symptom-based, categorical biomedical disorders within the individual. It diverted attention away from the social contexts that contribute to the causation of psychiatric problems and are crucial in their treatment.

## Why context diagnosis

DSM-III did not completely ignore the importance of social factors in psychiatric evaluation. It introduced the multiaxial diagnostic system (continued through DSM-III-R in 1987 and DSM-IV in 1994) explicitly including an Axis IV to document psychosocial and environmental problems that affect mental health conditions, their prognosis, and treatment (15). However, in practice, clinicians tended to focus almost exclusively on Axis I and II disorders, marginalizing contextual influences (16). DSM-5 (published in 2013) eliminated the useful multiaxial system entirely as part of its embrace of a simple minded and reductionist biological/medical model.

There are at least four reasons why it is important to bring context back into focus in mental health care. Firstly, despite 45 years of brain research, no single cause has been found in the brain for any disorder (17, 18). Nevertheless, many people believe that mental disorders are brain disorders, and this belief is associated with negative attitudes toward people with mental health diagnoses (19). Such stigma is a second reason to focus more attention on contextual determinants of mental suffering. A mental health label unjustifiably places the problems solely in the individual and can lead to exclusion (20) and self-stigma (21, 22), which in turn can result in diminished self-esteem, loss of hope and impaired social relationships (23). A third reason to diagnose and, where possible, treat contexts is the limited effectiveness of individual treatments for mental disorders. Leichsenring’s (24) recent umbrella review reported only modest effect sizes (0.34 for psychotherapy and 0.36 for medication). One reason for limited impact may be that clinicians focus too much on the patient and his or her DSM diagnosis and too little on the context in which problems are occurring. A recent viewpoint in JAMA explicitly states that requiring treatment for addiction or psychiatric issues before receiving housing would have created a barrier to the success of Housing First programs (25).

Finally, there is sufficient scientific evidence for the crucial role of context in the development, exacerbation, and maintenance of mental health problems. Kirbride et al. (26) provide an overview of social determinants of mental health problems, including poverty, negative childhood experiences, unemployment and job insecurity, debt, living in unsafe neighborhoods, homelessness or unstable housing, experiences of loss, loneliness, social isolation, lack of social support, exclusion, marginalization, and discrimination based on race, gender, or sexual orientation. Some of these factors may be difficult to influence from a clinical setting, but a recent Position Statement by the Canadian Psychiatric Association calls on mental health professionals to actively raise their voice and push back against sickening oppression, inequities and discrimination (27). In the clinical context it can be useful to identify and acknowledge intractable factors and recognize that they are very stressful. According to McMillen et al. (28), validating, that is, acknowledging the coherence or reasonableness of someone’s emotional or behavioral response in context, is a useful but underused clinical strategy.

## How to diagnose contexts

Following Bronfenbrenner (29), we define context as a set of nested environmental systems that influence human development and functioning, including the microsystem (e.g. daily interactions with partner/coworkers), mesosystem (e.g. the relationship between one’s workplace and family life), exosystem (e.g. local government policies affecting healthcare access), macrosystem (e.g. cultural values or national laws), and chronosystem (e.g. life transitions or historical events like a pandemic). Context diagnosis ensures that treatment is tailored to the possibilities in each of these systems in individual’s real-life context and not reduced to isolated behaviors and emotions. It offers a more comprehensive alternative to the DSM-IV’s Axis IV, which was often limited to superficial documentation of social factors without integrating them into treatment planning.

Context diagnosis explores the broad life context in which distress occurs. This includes a detailed contextual anamnesis, assessing aspects such as living conditions, work or educational setting, socio-economic situation, social relationships, life transitions, sources of stress, etcetera. In addition to this, the question “What matters to you” may open the door to holistic health solutions (30). The related social prescribing is an emerging contextual approach to addressing mental health challenges, involving explicitly non-medical referrals such as community groups and activities like community gardening, choirs, reading clubs, walking groups, and volunteering (31). **Table 1** offers a preliminary overview of some areas for contextual diagnostics, to be expanded in the future.

**Table 1:** Domains for context diagnosis, each of which could be assessed on two dimensions using a 1–10 scale (1): the degree to which the domain is influenceable, and (2) its priority for action. Taking these quantitative scores into account, clinicians work with individuals to make qualitative assessments of which contextual factors to address first.

Domain and examples	Examples of interventions
Past experiences -poverty -divorce -neglect -trauma	Validation (28)
Present living conditions -(impending) homelessness -quality of the house -neighbourhood	Housing condition interventions (32)
Work/school -quality -social safety -over- or underchallenging environment?	Diagnosing and treating organisations (33)
Socio-economic situation -income -debts	Financial support (34)
Facing discrimination/marginalisation	Problematize, pathologize and address the systems and narratives that discriminate (35) Actively push back against sickening inequity (27)
Feeling connected	Social prescribing (30)
What matters -past hobbies -present interests	Social prescribing (36)

In a concrete case, a young man may seek mental health care due to depressed moods. Alongside questions about mood (e.g. loneliness, isolation, sadness), the clinician explores his living situation (e.g. living with parents and younger sister), family dynamics (e.g. controlling parents), employment (e.g. low-paying, unfulfilling job), finances (e.g. unable to afford moving out), and past experiences (e.g. feeling unsafe, witnessing parental conflict). The clinician also inquiries about past (e.g. walking in nature) and present hobbies (e.g. diminished pleasure in all activities). Once sufficient information is gathered, the clinician formulates a contextual diagnosis, identifying contributing factors to the young man’s mental health issues. Often, this reveals that the problems are, at least partly, a logical reaction to life circumstances. The clinician can then validate unchangeable hardships and propose a treatment plan targeting modifiable contextual factors. In this case, interventions may include family therapy, referrals for financial aid and career services, and connecting the young man—given his loneliness and interest in nature—with a local walking group.

## Discussion

DSM diagnostics have benefited from promotion by interested parties, such as the pharmaceutical industry and biological psychiatry (37) and from the immense popularity of DSM-III. DSM diagnostics have been included in textbooks, courses, research funding, and treatment reimbursement. Consequently, we have a highly developed ability to psychopathologize and medicalize individuals, yet we lack a practical, sophisticated approach to diagnosing social or contextual deficiencies and linking them to interventions wherever possible.

More robust emphasis on contextual factors and the potential of context treatment and social prescribing as means to enhance treatment efficacy in mental healthcare settings is a promising avenue for future research and clinical applications. We offer three recommendations to provide greater emphasis on contextual factors and their corresponding solutions:

1. Develop some sort of contextual checklist or DSM-like handbook – like a Diagnostic and Statistical Manual of Contextual factors (DSC-I) - listing contextual categories that may contribute to mental issues and ways to address them (where possible). Like the DSM, a DSC might serve a clinical purpose in predicting prognosis, fostering clinical communication and planning (contextual) treatment, as well as a purpose in education, research funding and reimbursement. **Table 1** is a very preliminary starting point for a DSC.
2. Recognize the value of qualitative research. Context diagnosis and -intervention do not fit neatly within the conventional RCT framework which is considered the gold standard for providing the best possible evidence (38). RCTs are limited in evaluating complex, context-dependent interventions, as they rely on standardized protocols. Therefore, it is difficult for context work to receive the “evidence-based” label and (structural) funding. Qualitative designs offer greater flexibility, allowing for the exploration of broader theoretical frameworks. Furthermore, qualitative designs have been shown to facilitate more inclusive representation of marginalized groups, who are frequently overlooked in RCTs (39) and often undertreated (40).
3. Use a Stepped Diagnosis approach (41, 42). This cautious, context-sensitive approach aims to prevent overmedicalization without risking undertreatment. Rather than assigning psychiatric labels, it progresses through a series of minimal, proportional steps in collaboration with the person seeking support. These steps include exploring the personal and social context of distress, framing emotional distress as part of the normal human experience when appropriate, formulating advice, monitoring problems over time to see if they resolve naturally, and offering low-intensity, autonomy-promoting support such as bibliotherapy, self-help groups, or e-health tools. If problems persist, brief therapeutic support focused on practical problem-solving can be provided without a formal diagnosis. A psychiatric label should be considered if the distress is persistent, severe, and disabling, and then only in collaboration with the patient. This stepped approach prioritizes shared decision-making and allows for many contextual interventions.

Psychiatric Diagnosis and Context Diagnosis are complementary and clinicians - whether primary care or mental health- should be prepared to meet people’s needs in both dimensions.
